# Knowledge, attitudes, and practices related to health management among patients receiving intravesical instillation

**DOI:** 10.3389/fonc.2026.1879500

**Published:** 2026-08-03

**Authors:** Jingyang Wang, Siheng Li, Menghao Dong, Rong Ma, Rumeng Qin, Xiuxiu Tan

**Affiliations:** 1 Third Ward, Department of Urology, The First Affiliated Hospital of Anhui Medical University, Hefei, China; 2 School of Nursing, Anhui Medical University, Hefei, China; 3 Second Ward, Neurosurgery Department, The First Affiliated Hospital of Anhui Medical University, Hefei, China

**Keywords:** attitude, bladder irrigation, health management, knowledge, patients, practice

## Abstract

**Objective:**

This study aimed to investigate the knowledge, attitudes, and practices (KAP) related to health management among patients receiving intravesical instillation.

**Methods:**

This cross-sectional survey was conducted between July 1, 2025 and December 1, 2025. Patients receiving intravesical instillation were recruited using convenience sampling and completed an online, self-designed Chinese questionnaire distributed via Wenjuanxing and WeChat. The questionnaire collected sociodemographic characteristics and assessed three KAP domains.

**Results:**

A total of 422 valid questionnaires were included. Most participants were male (71.8%), with age groups of <60 years(27.3%), 60–69 years(39.8%), and ≥70 years (32.9%). Mean (SD) scores were 10.26(2.40) for knowledge(possible range: 0–15), 39.42 (6.90) for attitude (possible range: 12–60), and 39.39 (6.15) for practice (possible range: 10–50). SEM was conducted using a saturated path model with χ² = 0 and df = 0; therefore, the global fit indices were not interpreted as evidence of model adequacy. The SEM results showed significant direct effects of knowledge on practice (β=0.272, P<0.001) and attitude on practice (β=0.252, P<0.001), while the knowledge–attitude pathway was not significant (β=0.049, P = 0.317). Multivariate analysis showed that higher knowledge (OR = 1.291, 95%CI 1.170–1.424, P<0.001) and more positive attitudes (OR = 1.070, 95%CI 1.033–1.106, P<0.001) independently predicted better practice. Knowledge and attitude were both positively correlated with practice (r=0.306 and r=0.238, respectively; both P<0.001).

**Conclusion:**

Participants demonstrated suboptimal knowledge, neutral attitudes, and positive practices regarding health management during intravesical instillation. Improved practice was mainly driven by knowledge and attitudes rather than by a sequential knowledge-to-attitude effect. Proposed item-informed interventions, including clarification of regimen-related misconceptions, support for fear of recurrence and side-effect concerns, and training in simple coping behaviors, may help strengthen patient self-management and adherence, but their feasibility, acceptability, and effectiveness should be further evaluated in future implementation studies.

## Background

Bladder cancer is a common malignancy worldwide and represents a substantial public health burden, with approximately 573,000 new cases diagnosed globally in 2020 (1). Non–muscle-invasive bladder cancer (NMIBC) accounts for approximately 70–75% of newly diagnosed cases, representing the predominant clinical subtype at initial presentation (2). Although NMIBC is associated with favorable overall survival, recurrence remains common, with rates reaching up to 60% within the first year after transurethral resection of bladder tumor in the absence of appropriate adjuvant therapy (3). To reduce recurrence and delay disease progression, postoperative intravesical instillation therapy—including intravesical chemotherapy and Bacillus Calmette-Guérin (BCG) immunotherapy—has become a cornerstone of NMIBC management, with accumulating evidence demonstrating a significant reduction in tumor recurrence and its critical role in long-term disease control (4, 5). In China, bladder cancer has emerged as a major public health concern, with nearly 93,000 new cases estimated in 2022 according to GLOBOCAN 2022, underscoring the increasing disease burden and the need for effective long-term management strategies (6). Despite the widespread availability of intravesical therapy, suboptimal treatment adherence and inadequate long-term follow-up remain common in clinical practice, potentially undermining therapeutic effectiveness (7).

Intravesical instillation therapy typically requires repeated hospital visits, strict adherence to predefined treatment schedules, and prolonged surveillance protocols, making sustained patient engagement an essential component of treatment success in NMIBC (8). Nevertheless, treatment discontinuation and suboptimal adherence to guideline-recommended treatment and follow-up pathways remain prevalent, reflecting a persistent gap between recommended surveillance protocols and routine clinical practice (7, 9). In parallel, psychological distress among bladder cancer survivors, particularly anxiety and fear of recurrence, has been reported and may influence health-related behaviors and treatment-related decision-making (2, 10). Collectively, these findings suggest that effective NMIBC management extends beyond clinical interventions alone and warrants consideration of patient-related behavioral and psychosocial factors.

The Knowledge–Attitude–Practice (KAP) model provides a structured theoretical framework for assessing health-related behaviors by examining individuals’ disease-related knowledge, health beliefs, and behavioral practices (11). This model has been widely applied in studies of cancer prevention, demonstrating its utility in identifying modifiable behavioral determinants and informing targeted interventions (12, 13). Nevertheless, existing NMIBC-related research has predominantly focused on oncological outcomes, treatment efficacy, and guideline-based management (14, 15), whereas patient-centered evaluations of health management behaviors remain limited. Emerging evidence from Asian populations has highlighted the importance of treatment adherence, guideline compliance, and psychological burden among patients with bladder cancer or NMIBC. For example, Japanese real-world studies have examined maintenance BCG use and guideline compliance in NMIBC management (16, 17), while Chinese studies have reported substantial fear of cancer recurrence and explored psychosocial nursing interventions among patients with bladder cancer (10, 18). However, these studies have mainly focused on clinical management, adherence, or psychological outcomes rather than systematically examining knowledge, attitudes, and practices related to health management among patients receiving intravesical instillation. Therefore, evidence from Asian or Chinese populations remains limited, and the potential roles of cultural context and health information–seeking behaviors in shaping patient perceptions and practices remain insufficiently explored.

Therefore, the present study aims to assess the knowledge, attitudes, and practices related to health management among patients receiving intravesical instillation therapy using a KAP-based survey. The study further examines knowledge gaps, attitudinal barriers, and practice patterns associated with treatment adherence and follow-up, as well as relevant demographic, clinical, and information-related factors. The findings may provide empirical data to inform patient-oriented health management and education strategies for NMIBC.

## Methods

### Study design and participants

This cross-sectional study was conducted from July 1, 2025 to December 1, 2025. Patients receiving intravesical instillation were recruited using a convenience sampling approach at the First Affiliated Hospital of Anhui Medical University. Eligible participants met the following inclusion criteria (1): age between 18 and 91 years (2); clinicopathologically confirmed bladder cancer with postoperative intravesical instillation therapy (3); provision of written informed consent and voluntary participation in the study; and (4) adequate self-care ability and clear verbal communication. Exclusion criteria included refusal to participate, questionnaire completion time exceeding 1800 seconds, and questionnaires containing abnormal or implausible values in key items. The study protocol was approved by the Institutional Ethics Committee of the First Affiliated Hospital of Anhui Medical University, and informed consent was obtained from all participants prior to enrollment.

### Procedures

A self-designed questionnaire was employed in this study. Its development was informed by disease-specific clinical guidance and validated psychological assessment instruments. The knowledge domain was developed with reference to the AUA/SUO Guideline on the Diagnosis and Treatment of Non-Muscle Invasive Bladder Cancer and the 2022 Edition of the Chinese Guidelines for the Diagnosis and Treatment of Urological and Andrological Diseases (19). The attitude and practice domains were developed with reference to relevant dimensions from validated cancer-related psychological and symptom assessment instruments, including the Fear of Cancer Recurrence Inventory and the Patient Health Questionnaire-9 (20, 21), particularly for items related to fear of recurrence, emotional burden, coping, and health management behaviors. The questionnaire was further refined through a qualitative expert review process rather than a quantitative content validity index assessment. Three experts participated in the review, including two deputy chief nurses with clinical experience in urological nursing and intravesical instillation care and one deputy chief physician specializing in urology. The experts independently reviewed the questionnaire for clinical relevance, clarity, comprehensiveness, wording accuracy, and appropriateness for patients receiving intravesical instillation. Their feedback was then discussed by the research team, and revisions were made by consensus. The revised questionnaire subsequently underwent pilot testing. In the pilot phase, 41 questionnaires were collected, of which 40 were deemed valid after excluding responses lacking informed consent or with a completion time of less than 90 seconds. Based on patient feedback during the pilot phase, the wording of two knowledge-domain items was simplified to improve comprehension among older patients, while the core content and overall item structure were retained. The overall internal consistency of the questionnaire was acceptable, with a Cronbach’s α coefficient of 0.861; the corresponding values for the knowledge, attitude, and practice domains were 0.796, 0.815, and 0.768, respectively.

The final questionnaire was administered in Chinese and comprised four domains. The basic information domain included 15 items, while the knowledge, attitude (health belief), and practice domains consisted of 15, 12, and 10 items, respectively. In the knowledge domain, responses were scored as 1 point for correct answers and 0 points for incorrect or uncertain answers, yielding a possible total score of 0–15. Both the attitude and practice domains adopted a five-point Likert scale, with response options ranging from very positive (5 points) to very negative (1 point) according to item polarity. The attitude domain had a possible score range of 12–60, and the practice domain ranged from 10 to 50. Based on the possible score ranges, knowledge levels were categorized as insufficient (0–9), moderate (10–12), or sufficient (13–15), corresponding approximately to <60%, 60%–79%, and ≥80% of the maximum knowledge score, respectively. Attitudes were classified as negative (12–30), neutral (31–45), or positive (46–60), and practice behaviors were defined as negative (10–25), moderate (26–37), or positive (38–50).

For questionnaire administration, participants were recruited using convenience sampling. Data were collected through an online survey created on the Wenjuanxing platform and distributed via WeChat using a QR code. Participants completed the questionnaire by scanning the code. To ensure data quality, each IP address was permitted a single submission, and all items were set as mandatory. Completed questionnaires were reviewed by the research team for completeness, internal consistency, and logical plausibility. Responses were excluded if the completion time was less than 90 seconds, if response patterns were illogical, or if identical options were selected for all items within any single KAP domain.

### Sample size calculation

The minimum sample size for this cross-sectional survey was calculated using the standard formula for estimating a population proportion: n = Z²p(1−p)/d². Because no prior estimate of the proportion of adequate health management practice among patients receiving intravesical instillation was available, p was set at 0.50 to obtain the maximum required sample size. With a two-sided α level of 0.05, Z = 1.96, and an allowable margin of error of 0.05, the calculated minimum sample size was 384.16, which was rounded up to 385 participants. In addition, the adequacy of the sample size for structural equation modeling was evaluated according to commonly used SEM sample size recommendations. Specifically, a sample size greater than 200 is generally considered acceptable for SEM, and at least 10 observations per estimated parameter are often recommended. Because the present path model included no more than 20 estimated parameters, at least 200 participants were required for SEM. Therefore, the final sample of 422 valid questionnaires exceeded both the minimum requirement for the cross-sectional survey and the commonly used SEM sample size requirement.

### Statistical analyses

Data analysis was performed using R software (version 4.3.2). Continuous variables were first assessed for normality. Data conforming to a normal distribution are presented as means with standard deviations, whereas non-normally distributed data are summarized as medians with the 25th and 75th percentiles. Categorical variables are expressed as numbers and percentages. For comparisons between two groups, independent-samples t tests were applied to normally distributed continuous variables, while the Wilcoxon–Mann–Whitney test was used when normality assumptions were violated. When comparing three or more groups, one-way analysis of variance was employed for normally distributed data with homogeneity of variance, and the Kruskal–Wallis test was used for non-normally distributed data. Associations between domain scores were examined using Pearson correlation coefficients for normally distributed variables and Spearman rank correlation coefficients for variables that did not meet normality assumptions. Knowledge, attitude, and practice domain scores were treated as dependent variables in univariate and multivariate regression analyses to evaluate their associations with demographic characteristics. For regression analyses, outcomes were dichotomized using the median value of each domain score as the cut-off. Variables with a P value <0.10 in univariate analyses were subsequently entered into multivariate models using the glm function from the core stats package in R. All P values were reported to three decimal places, and a two-sided P value <0.05 was considered statistically significant. Within the knowledge–attitude–practice theoretical framework, structural equation modeling was applied to examine the potential mediating role of attitude in the relationship between knowledge and practice and to estimate both direct and indirect effects. Given that the primary objective was to examine domain-level associations among knowledge, attitudes, and practices, and because the knowledge domain consisted of dichotomously scored items covering heterogeneous disease-related content, an observed-variable path model was used rather than a latent-variable SEM. Because the specified path model was saturated/just-identified, rather than over-identified, with zero degrees of freedom, global fit indices could not be used to evaluate model fit. Instead, interpretation of the SEM results focused on the estimated path coefficients, confidence intervals, and corresponding P values. Structural equation modeling was performed using the Structural Equation Modeling (SEM) Builder module in Stata software (version 18.0).

## Results

### Questionnaire quality

Initially, this study collected a total of 438 questionnaires. The following samples were excluded: 1) Three cases without informed consent; 2) Three cases with abnormal age values; 3) Five cases with abnormal height values and two cases with abnormal weight values; 4) One case with an abnormal value for the number of recurrences; and 5) Two cases with a response time of less than 90s. The criteria for excluding outliers in baseline physical characteristics and disease recurrence variables are shown in **Supplementary Table 1**. Ultimately, 422 valid questionnaires were retained, with a validity rate of 96.35%. In the formal survey, the overall scale demonstrated acceptable internal consistency, with an overall Cronbach’s α coefficient of 0.8457. The Cronbach’s α coefficients were 0.6199 for the knowledge domain, 0.8228 for the attitude domain, and 0.7870 for the practice domain. The relatively low Cronbach’s α coefficient for the knowledge domain may be partly attributable to the dichotomous scoring of knowledge items and the greater heterogeneity of the formal survey sample compared with the pilot sample. Therefore, the internal consistency of the knowledge domain should be interpreted with caution. practice To further evaluate the psychometric properties of the questionnaire, factor analysis was additionally performed. The Kaiser–Meyer–Olkin (KMO) value of the overall scale was 0.8118, and Bartlett’s test of sphericity was statistically significant (χ² = 5408.293, df = 666, P < 0.001) (**Supplementary Table 2**). Confirmatory factor analysis showed acceptable model fit, with RMSEA = 0.073, SRMR = 0.058, TLI = 0.893, and CFI = 0.904 (**Supplementary Table 3**). The CFA path coefficients and factor loadings are presented in **Supplementary Table 4**.

### Demographic information on participants and KAP scores

The study included 422 patients receiving intravesical instillation. Most participants were male (71.8%), with a mean age distribution across groups <60 years (27.3%), 60–69 years (39.8%), and ≥70 years (32.9%). Nearly half had a BMI between 18.50 and 23.99 (49.1%), and farmers constituted the largest occupational group (42.9%). Their mean (SD) knowledge, attitude, and practice scores were 10.26 (2.40), 39.42 (6.90), and 39.39 (6.15), respectively. Knowledge scores differed significantly by BMI category (P < 0.001), occupation (P = 0.003), educational level (P = 0.009), and the number of completed intravesical instillation treatments (P < 0.001). Practice scores showed significant associations with occupation (P = 0.001), educational level (P < 0.001), time since surgery (P < 0.001), and the number of completed treatments (P = 0.002) (**Table 1**).

**Table 1:** Demographic characteristics and KAP scores.

Variables	N (%)	Knowledge mean (SD)	P	Attitude mean (SD)	P	Practice mean (SD)	P
Total score	422 (100.0)	10.26 (2.40)		39.42 (6.90)		39.39 (6.15)	
Gender			0.459		0.475		0.758
Male	303 (71.8)	10.31 (2.35)		39.57 (6.75)		39.45 (6.10)	
Female	119 (28.2)	10.12 (2.54)		39.03 (7.30)		39.24 (6.31)	
Age			0.259		0.734		0.994
<60	115 (27.3)	10.21 (2.46)		39.10 (5.88)		39.42 (5.33)	
60-69	168 (39.8)	10.48 (2.10)		39.73 (6.82)		39.41 (6.14)	
≥70	139 (32.9)	10.03 (2.66)		39.31 (7.77)		39.35 (6.80)	
BMI			<0.001		0.209		0.944
<18.50	16 (3.8)	11.31 (2.09)		39.62 (5.82)		39.44 (5.78)	
18.50-23.99	207 (49.1)	10.17 (2.33)		38.71 (7.19)		39.25 (6.42)	
24.00-27.99	175 (41.5)	10.51 (2.15)		40.07 (6.54)		39.47 (5.98)	
≥28.00	24 (5.7)	8.42 (3.74)		40.62 (7.38)		40.00 (5.58)	
Occupation			0.003		0.678		0.001
Farmer	181 (42.9)	9.83 (2.51)		39.39 (7.01)		38.02 (6.47)	
Worker	51 (12.1)	10.59 (2.32)		38.39 (6.47)		39.47 (6.19)	
Retired	131 (31.0)	10.79 (2.02)		39.66 (7.28)		40.69 (5.62)	
Other	59 (14)	10.07 (2.68)		39.85 (6.12)		40.66 (5.43)	
Educational level			0.009		0.140		<0.001
Primary school or below	143 (33.9)	9.78 (2.75)		39.32 (7.14)		37.65 (6.86)	
Junior high school	141 (33.4)	10.30 (2.28)		38.72 (6.83)		39.89 (5.63)	
Senior high school/technical secondary school	71 (16.8)	10.92 (2.10)		39.41 (7.41)		40.07 (5.76)	
Associate degree or above	67 (15.9)	10.48 (1.94)		41.10 (5.76)		41.33 (5.12)	
Time since surgery			0.055		0.175		<0.001
<6 months	155 (36.7)	10.30 (2.71)		40.24 (6.76)		41.14 (5.62)	
6–12 months	145 (34.4)	9.91 (2.22)		38.88 (7.29)		38.90 (6.17)	
>1 year	122 (28.9)	10.61 (2.14)		39.01 (6.56)		37.75 (6.26)	
Number of completed intravesical instillation treatments			<0.001		0.796		0.002
<10 times	147 (34.8)	10.30 (2.58)		39.27 (6.70)		40.81 (6.25)	
10–19 times	181 (42.9)	9.81 (2.39)		39.67 (7.44)		38.71 (5.98)	
≥20 times	94 (22.3)	11.04 (1.88)		39.15 (6.16)		38.49 (6.00)	
Recurrence			0.512		0.064		0.050
Yes	89 (21.1)	10.40 (2.48)		38.21 (5.97)		38.26 (6.31)	
No	333 (78.9)	10.22 (2.38)		39.74 (7.11)		39.69 (6.08)	

Mean (SD) values in this table are purely descriptive and were not used as cut-off points for subsequent regression analyses.

### Distribution of responses to knowledge, attitude, and practice

The distribution of knowledge dimensions showed that the three questions with the highest number of participants choosing the ‘Uncertain’ option were ‘Traditional Chinese medicine remedies can replace standard intravesical instillation therapy.’ (K9) with 52.84%, ‘Gemcitabine intravesical instillation can be used in patients who fail BCG therapy.’ (K12) with 51.42%, and ‘The total duration of intravesical chemotherapy is usually shorter than that of BCG therapy (e.g., intermediate-risk patients receiving chemotherapy may require only 6–12 weeks, whereas BCG therapy requires maintenance for 1–3 years).’ (K14) with 50.95% (**Supplementary Table 5**). The complete item wording and response distributions for the knowledge, attitude, and practice domains are provided in **Supplementary Tables 5**–**S7**.

Responses to the attitude dimension showed that a majority (51.90%) agreed that concerns about side effects affect their willingness to continue long-term therapy (A3), with an additional 28.20% remaining neutral. Concurrently, fear of disease recurrence manifested as severe anxiety, with 39.58% reporting sleep difficulties (A4) and 37.67% experiencing palpitations (A5) due to this worry. This anxiety may be exacerbated by catastrophic interpretations of recurrence, as 27.96% viewed recurrence as indicative of complete treatment failure (A6). Critically, these concerns translated into substantial negative emotional states, over one-third of respondents reported frequent low mood (36.49%, A10), a loss of future confidence (32.46%, A11), and an inability to cope with daily disease-related stress (31.75%, A12) (**Supplementary Table 6**).

Responses to the practice dimension showed that only 17.77% always shared treatment-related stress with family members (P9), only 20.38% always relieved anxiety through deep-breathing exercises (P8), only 24.41% always reduced psychological stress by adjusting my perspective on the disease and focusing on positive aspects. (P10) (**Supplementary Table 7**).

### Correlation analysis

Spearman correlation analysis showed that there were positive correlations between knowledge and practice (r = 0.306, P < 0.001) as well as between attitude and practice (r = 0.238, P < 0.001). The correlation between knowledge and attitudes was not statistically significant (**Table 2**).

**Table 2:** Correlation analysis.

Spearman	Knowledge	Attitude	Practice
Knowledge	1.000		
Attitude	0.082 (P = 0.093)	1.000	
Practice	0.306 (P<0.001)	0.238 (P<0.001)	1.000

### Univariate and multivariate analysis

The median of the knowledge, attitude, and practice scores were used as the cut-off value for each dimension to divided the groups, and the number of participants above the cut-off value were 213 (50.5%), 216 (51.2%), and 218 (51.7%), respectively. In the multivariate analysis, a BMI of 28.00 or higher was associated with a lower level of knowledge at a borderline level of significance (OR = 0.255, 95% CI: [0.065-0.997], P = 0.050). A history of non-recurrence was a significant independent predictor of a positive attitude (OR = 0.575, 95% CI: [0.356-0.929], P = 0.024). Higher knowledge (OR = 1.291, 95% CI: [1.170-1.424], P < 0.001) and attitude (OR = 1.070, 95% CI: [1.033-1.106], P < 0.001) scores were both strong independent predictors of good practice. Furthermore, a higher educational level was significantly associated with better practice. Compared to a primary school education or below, patients who had completed senior high school or technical secondary school (OR = 2.471, 95% CI: [1.209-5.048], P = 0.013) and those with an associate degree or above (OR = 3.155, 95% CI: [1.415-7.039], P = 0.005) were more likely to have good practice (**Table 3**).

**Table 3:** Univariate and multivariate analysis.

Variables	Univariate analysis		Multivariate analysis	
	OR (95%CI)	P	OR (95%CI)	P
Outcome: Knowledge				
Gender				
Male				
Female	0.951 (0.622,1.455)	0.818		
Age				
<60				
60-69	1.245 (0.774,2.006)	0.366		
≥70	0.953 (0.581,1.563)	0.847		
BMI				
<18.50				
18.50-23.99	0.509 (0.168,1.422)	0.207	0.530 (0.181,1.549)	0.246
24.00-27.99	0.819 (0.268,2.306)	0.711	0.831 (0.281,2.459)	0.739
≥28.00	0.247 (0.061,0.913)	0.041	0.255 (0.065,0.997)	0.050
Occupation				
Farmer				
Worker	1.957 (1.045,3.734)	0.038	1.712 (0.884,3.315)	0.111
Retired	1.494 (0.952,2.353)	0.082	1.343 (0.842,2.142)	0.216
Other	1.398 (0.776,2.530)	0.265	1.373 (0.746,2.527)	0.309
Educational level				
Primary school or below				
Junior high school	1.218 (0.765,1.943)	0.407		
Senior high school/technical secondary school	1.383 (0.782,2.458)	0.267		
Associate degree or above	1.169 (0.653,2.094)	0.599		
Time since surgery				
<6 months				
6–12 months	0.668 (0.423,1.052)	0.082	0.708 (0.368,1.364)	0.302
>1 year	0.964 (0.599,1.552)	0.880	0.539 (0.250,1.162)	0.115
Number of completed intravesical instillation treatments				
<10 times				
10–19 times	0.688 (0.444,1.065)	0.094	0.801 (0.424,1.511)	0.492
≥20 times	1.465 (0.867,2.493)	0.156	2.088 (0.894,4.879)	0.089
Recurrence				
Yes				
No	1.126 (0.705,1.803)	0.620		
Outcome: Attitude				
**Attitude**	Univariate analysis		Multivariate analysis	
	OR (95%CI)	P	OR (95%CI)	P
**Knowledge**	0.967 (0.893,1.047)	0.411	0.958 (0.882,1.040)	0.304
Gender				
Male				
Female	1.004 (0.657,1.536)	0.985		
Age				
<60				
60-69	1.398 (0.869,2.256)	0.168		
≥70	1.379 (0.841,2.270)	0.204		
BMI				
<18.50				
18.50-23.99	0.550 (0.181,1.537)	0.264		
24.00-27.99	0.713 (0.234,2.005)	0.529		
≥28.00	0.600 (0.158,2.152)	0.438		
Occupation				
Farmer				
Worker	0.868 (0.462,1.618)	0.657	0.847 (0.450,1.595)	0.607
Retired	1.507 (0.959,2.379)	0.077	1.553 (0.976,2.472)	0.063
Other	0.955 (0.528,1.720)	0.877	0.946 (0.523,1.712)	0.854
Educational level				
Primary school or below				
Junior high school	0.731 (0.457,1.166)	0.189		
Senior high school/technical secondary school	1.237 (0.699,2.202)	0.466		
Associate degree or above	1.611 (0.895,2.943)	0.116		
Time since surgery				
<6 months				
6–12 months	0.903 (0.573,1.420)	0.658		
>1 year	0.890 (0.553,1.431)	0.631		
Number of completed intravesical instillation treatments				
<10 times				
10–19 times	1.230 (0.796,1.904)	0.352		
≥20 times	0.998 (0.594,1.676)	0.995		
Recurrence				
Yes				
No	0.577 (0.357,0.925)	0.023	0.575 (0.356,0.929)	0.024
Outcome: Practice				
**Knowledge**	1.280 (1.169,1.401)	<0.001	1.291 (1.170,1.424)	<0.001
**Attitude**	1.046 (1.017,1.076)	0.002	1.070 (1.033,1.106)	<0.001
Gender				
Male				
Female	0.978 (0.640,1.496)	0.918		
Age				
<60				
60-69	0.741 (0.459,1.191)	0.217		
≥70	1.093 (0.665,1.798)	0.725		
BMI				
<18.50				
18.50-23.99	0.849 (0.293,2.362)	0.753		
24.00-27.99	0.787 (0.270,2.204)	0.648		
≥28.00	0.919 (0.252,3.294)	0.897		
Occupation				
Farmer				
Worker	0.980 (0.519,1.829)	0.948	0.628 (0.303,1.301)	0.210
Retired	2.092 (1.326,3.324)	0.002	1.226 (0.684,2.196)	0.494
Other	2.021 (1.116,3.721)	0.022	1.367 (0.637,2.934)	0.422
Educational level				
Primary school or below				
Junior high school	1.621 (1.014,2.604)	0.044	1.681 (0.970,2.913)	0.064
Senior high school/technical secondary school	2.386 (1.340,4.307)	0.003	2.471 (1.209,5.048)	0.013
Associate degree or above	3.651 (1.985,6.912)	<0.001	3.155 (1.415,7.039)	0.005
Time since surgery				
<6 months				
6–12 months	0.434 (0.272,0.689)	<0.001	0.512 (0.249,1.053)	0.069
>1 year	0.397 (0.243,0.645)	<0.001	0.462 (0.198,1.076)	0.074
Number of completed intravesical instillation treatments				
<10 times				
10–19 times	0.478 (0.305,0.744)	0.001	0.800 (0.397,1.613)	0.533
≥20 times	0.436 (0.256,0.738)	0.002	0.598 (0.238,1.500)	0.273
Recurrence				
Yes				
No	0.633 (0.393,1.013)	0.058	0.600 (0.342,1.051)	0.074

The median value of each domain score was used as the cut-off point to dichotomize participants into higher and lower score groups. For the knowledge domain, scores ≥11 were classified as higher knowledge and scores ≤10 as lower knowledge. For the attitude domain, scores ≥40 were classified as more positive attitude and scores ≤39 as less positive attitude. For the practice domain, scores ≥40 were classified as better practice and scores ≤39 as poorer practice.

### SEM analysis

**Figure 1:**
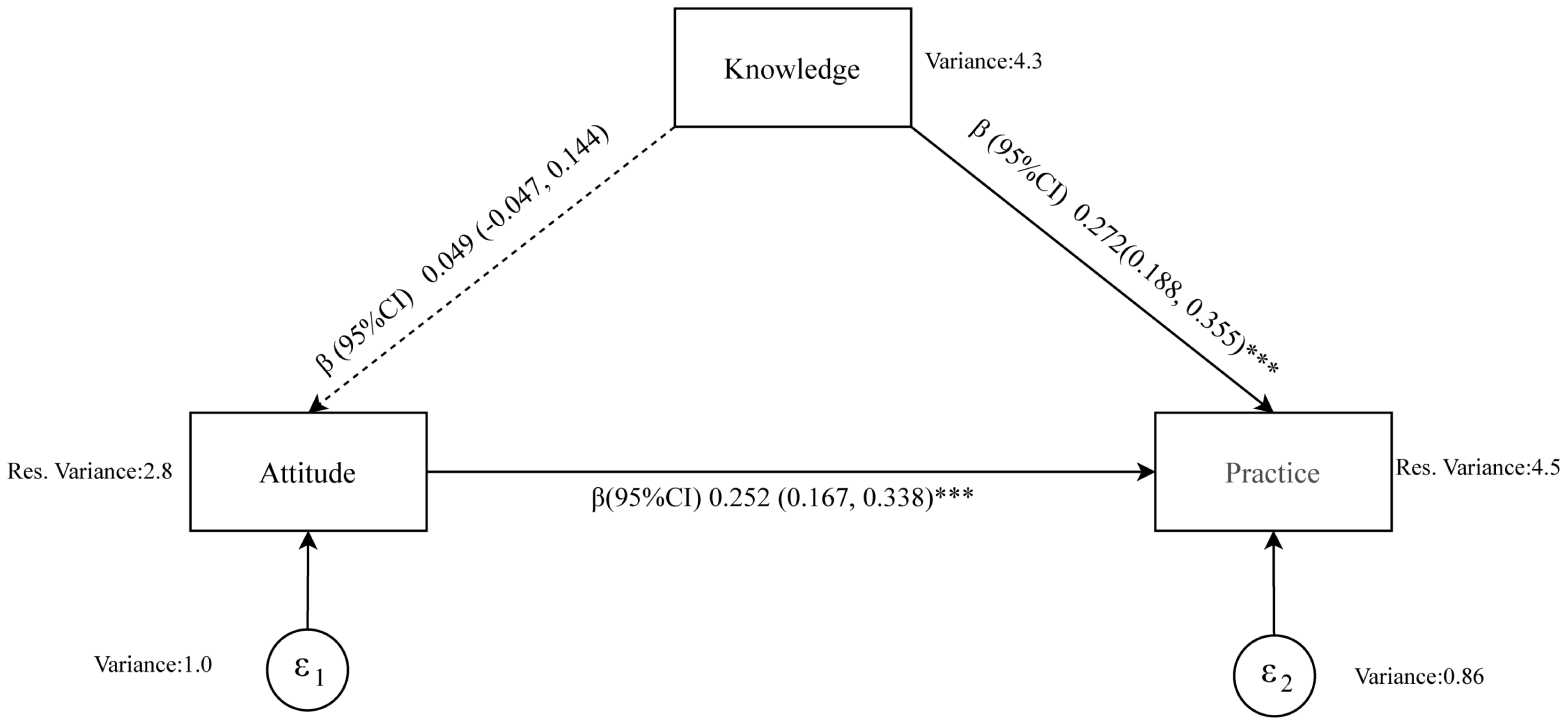
Structural equation modeling (SEM) path diagram for the Knowledge-Attitude-Practice (KAP) model. Solid arrows represent statistically significant pathways (P < 0.001), whereas the dashed arrow represents a non-significant pathway (P ≥ 0.05). Values along the pathways indicate the standardized path coefficients (β) with their corresponding 95% confidence intervals (CIs). The numbers enclosed within or adjacent to the variable boxes (4.3, 2.8, and 4.5) denote the total variances for the exogenous variable (Knowledge) and residual variances for the endogenous variables (Attitude and Practice), respectively. ϵ 1 and ϵ 2 represent the error/residual terms for Attitude and Practice, with their scaling or residual variance weights indicated by 1.00 and 0.86. ***P < 0.001.

The measurement model detailing the factor loadings of all questionnaire items (k1–k15, a1–a12, and p1–p10) on their respective latent constructs (Knowledge, Attitude, and Practice), along with their residual variances (ϵ1–ϵ37), is presented in **Supplementary Figure 1**. Subsequently, the specified SEM was a saturated/just-identified path model, rather than an over-identified model, with χ² = 0 and df = 0. Accordingly, the global fit indices shown in **Supplementary Table 8**, including RMSEA = 0.000, SRMR = 0.000, TLI = 1.000, and CFI = 1.000, were not interpreted as evidence of superior model fit. Instead, the SEM results were interpreted based on the estimated path coefficients and their statistical significance. The direct effect of knowledge on practice was significant (β = 0.272, P < 0.001), as was the direct effect of attitude on practice (β = 0.252, P < 0.001). However, the direct effect of knowledge on attitude was not statistically significant (β = 0.049, P = 0.317), and the indirect effect of knowledge on practice through attitude was also not statistically significant (β = 0.012, P = 0.323) (**Table 4**, **Figure 1**).

**Table 4:** Analysis of direct and indirect effects.

Model paths		Total effects		Direct Effect		Indirect effect	
		β(95%CI)	P	β(95%CI)	P	β(95%CI)	P
Attitude							
	Knowledge	0.049 (-0.047, 0.144)	0.317	0.049 (-0.047, 0.144)	0.317		
Practice							
	Knowledge	0.284 (0.198, 0.370)	<0.001	0.272 (0.188, 0.355)	<0.001	0.012 (-0.102, 0.036)	0.323
	Attitude	0.252 (0.167, 0.338)	<0.001	0.252 (0.167, 0.338)	<0.001		

## Discussion

Patients receiving intravesical instillation demonstrated suboptimal disease-related knowledge, predominantly neutral attitudes, and positive practice behaviors. practice behavior Future clinical management may incorporate structured, risk-stratified education programs integrated into routine intravesical instillation care, combined with brief, potentially feasible psychosocial support strategies and validated digital health tools, to simultaneously address knowledge gaps and attitudinal barriers and thereby promote sustainable, high-quality self-management behaviors.

The absence of a supported knowledge-to-attitude pathway merits careful interpretation because many KAP applications assume a sequential progression from information acquisition to attitudinal change and then to behavior. In the present study, however, knowledge and attitudes appeared to function as partially independent determinants of practice rather than as a linear causal chain. This finding can be better understood through broader behavioral theories (22). From the perspective of the Health Belief Model, patients’ health behaviors may be driven not only by factual knowledge but also by perceived susceptibility, perceived severity, perceived benefits, perceived barriers, and cues to action (23). For patients receiving intravesical instillation, fear of recurrence, awareness of cancer severity, repeated exposure to hospital-based treatment, and direct clinician instructions may serve as strong cues to action and may promote adherence even when disease-specific knowledge remains incomplete. In other words, patients may maintain procedural adherence because they perceive bladder cancer recurrence and treatment interruption as serious threats, rather than because knowledge has first been translated into stable positive attitudes. Similarly, the Theory of Planned Behavior suggests that behavior is shaped by attitudes, subjective norms, and perceived behavioral control (22). In this clinical context, trust in clinicians, family expectations, structured treatment schedules, and perceived control over follow-up behaviors may directly support practice, while emotional distress or uncertainty may prevent attitudes from becoming uniformly positive. Therefore, the non-significant knowledge-to-attitude pathway does not necessarily contradict the KAP framework; rather, it suggests that, in a high-threat oncology context, affective risk appraisal, clinical cues, perceived control, and social norms may operate alongside knowledge to shape health management behaviors. The response distributions clarify why knowledge and attitudes may decouple in this population. Uncertainty clustered around comparative treatment regimens, treatment duration, escalation options after inadequate response, and the role of nonstandard or alternative approaches, which are cognitively demanding topics that often require individualized risk stratification and nuanced counseling. Evidence from real-world intravesical therapy studies indicates that treatment delivery varies across clinical settings and that deviations from recommended maintenance schedules remain common, creating an informational environment where patients may receive inconsistent messages over time (16, 24). At the same time, participants largely endorsed trust in clinicians over online information while also reporting mixed confidence in evaluating digital content, a combination that may reduce misinformation driven behaviors but can leave knowledge gaps unfilled when clinic encounters are brief or when patients hesitate to ask clarifying questions (25, 26). The multivariate association between education and practice supports this reading by suggesting that patients with greater educational resources may navigate regimen complexity and information verification more effectively, even when attitudes remain only modestly positive (25). These findings should also be interpreted within the Asian and Chinese care context. Previous Chinese studies on bladder cancer patients have emphasized fear of recurrence and the potential value of psychosocial nursing interventions (10, 18), suggesting that emotional burden may strongly shape patient perceptions and self-management needs. In addition, Asian real-world NMIBC studies from Japan have shown that maintenance treatment patterns and guideline compliance remain important issues in long-term management (16, 17). Together, these findings support the need for culturally and clinically contextualized health education that addresses both regimen-specific knowledge and psychosocial barriers.

The attitudinal profile points to a high emotional load that plausibly competes with learning and reframes decision making. Many respondents reported worry about recurrence, sleep related disturbance, somatic anxiety symptoms, and depressed mood, alongside concerns that side effects could undermine willingness to continue long term therapy, indicating that affective threat and treatment burden remained salient even when patients endorsed the value of following medical advice (27). Prior bladder cancer studies have shown that distress is related not only to prognosis uncertainty but also to repeated invasive procedures and symptom vigilance, which can sustain anticipatory anxiety across follow-up cycles (27, 28). The multivariate association between recurrence history and attitudes complements this interpretation by indicating that clinical experience with recurrence may shape beliefs and emotional responses independently of knowledge level, consistent with evidence that lived experiences recalibrate perceived threat even when informational content remains stable (27).

Practice responses revealed a clinically important split between high adherence to the procedural backbone of intravesical care and weaker uptake of coping oriented behaviors. Patients commonly reported timely attendance for instillation and engagement with recommended monitoring and follow up behaviors, yet far fewer reported routinely using simple anxiety reduction strategies or sharing treatment related stress with family members. Consistent with prior evidence on supportive care in bladder cancer, adherence to scheduled cancer treatments can remain strong when care pathways are explicit and reinforced by clinical teams, whereas psychosocial self-management may lag because it is less protocolized, less frequently taught with demonstration, and sometimes constrained by stigma or norms of emotional restraint (28). This pattern also fits the SEM structure in which attitudes had a direct relationship with practice, implying that emotional burden and beliefs about coping may influence the psychosocial components of practice even when procedural adherence remains intact (27, 29).

Several group differences observed in unadjusted comparisons require integration with the multivariate findings to avoid over interpretation. Differences in knowledge across body weight categories, occupation, education, and treatment exposure may initially appear to reflect broad sociodemographic gradients. However, after adjustment, only BMI ≥28.00 retained a borderline association with lower knowledge (OR = 0.255, 95% CI: 0.065–0.997, P = 0.050). It is worth noting that this association only reached borderline statistical significance and should therefore be interpreted with caution. Evidence from cancer care research indicates that obesity may correlate with lower health literacy and higher comorbidity burden in some populations, which may complicate comprehension and retention of regimen details; however, given the borderline significance observed in the present study, this explanation remains tentative (25). The attenuation of occupation and treatment exposure after adjustment may indicate that these variables operate through more proximal determinants such as educational opportunity, clinician communication time, or symptom burden, rather than exerting independent effects (26, 28). In contrast, educational attainment remained associated with better practice in multivariate analysis, which aligns with research suggesting that education supports navigation of complex care instructions, structured information seeking, and sustained follow up behaviors (17, 25).

The combined correlation, SEM, and multivariate findings also inform how overall KAP status should be understood. Knowledge levels appeared low relative to the instrument’s possible range, while attitudes clustered around neutrality despite substantial endorsement of clinician guidance, and practices appeared comparatively stronger, at least for core treatment behaviors. Prior studies of intravesical therapy have indicated that routine compliance may be supported by repeated contact with health professionals and clear scheduling, even when patients remain uncertain about comparative regimens or long-term planning (16, 30). At the same time, evidence indicates that intolerance and adverse effects contribute to treatment interruption in non-muscle-invasive bladder cancer, underscoring why attitudes and symptom-related fear can matter for sustaining practice over time practice (16, 24). The present model’s parallel paths imply that strengthening practice may require two distinct levers: improving regimen comprehension and addressing emotional barriers, rather than expecting one to automatically resolve the other (27, 28).

Recommendations should follow directly from the specific weaknesses suggested by item distributions and the model structure. Education should prioritize the domains where uncertainty concentrated, particularly treatment duration expectations, differences between intravesical approaches, appropriate responses to common side effects, and the limits of nonstandard alternatives, delivered using short modules timed to key clinical transitions such as therapy initiation, regimen changes, or approaching maintenance phases. Real-world evidence on maintenance therapy indicates that practice patterns often diverge from recommendations; therefore, counseling that anticipates variation and explains the rationale for treatment duration may reduce confusion and support persistence when schedules extend (16). Communication strategies should incorporate teach back and scenario based questions to verify understanding of regimen contingencies, especially for patients with lower educational attainment, given the persistent association between education and practice after adjustment (25). Psychosocial components need equal specificity: brief clinician guided coping sessions can be embedded into routine visits, focusing on one or two skills that patients can rehearse in clinic such as paced breathing or structured worry postponement, with follow up prompts at subsequent instillations to encourage adoption. Meta-analytic evidence has shown that psychological interventions can reduce fear of recurrence and that concise, process-focused approaches may produce measurable benefits, supporting feasibility within busy outpatient pathways (31). Bladder cancer specific programs that frame coping as part of treatment adherence, including structured gratitude or reframing exercises, may fit patient expectations for practical guidance while addressing the prominent distress signals seen in attitudinal responses (18, 29). Digital tools can extend these interventions between visits by offering validated education, symptom logging, and tailored alerts that prompt timely clinician contact, although implementation should account for variable digital literacy and provide low burden interfaces and family supported onboarding where appropriate (26, 32).

Several limitations of this study should be acknowledged. First, the cross-sectional design precludes causal inference among knowledge, attitudes, and practices, and the observed associations and SEM pathways should therefore be interpreted as correlational rather than directional; however, the use of multivariate analysis and SEM helped to control for key confounders and to strengthen the internal consistency of the findings. Second, participants were recruited from a single tertiary hospital using convenience sampling and completed the questionnaire online through WeChat and Wenjuanxing, which introduced a systematic self-selection bias that could not be statistically corrected retrospectively. In addition, the sample was predominantly male and included a substantial proportion of older patients, which may reflect the clinical profile of bladder cancer to some extent but also limits the representativeness of the findings. Patients who had better access to smartphones, higher digital literacy, stronger health engagement, or greater willingness to participate in online surveys may have been more likely to respond. Conversely, patients with limited smartphone use and those from socioeconomically disadvantaged or less digitally connected regions may have been underrepresented. Therefore, the findings should be generalized to the broader population of Chinese patients with NMIBC or those receiving intravesical instillation with caution, and multicenter studies with more diverse samples are needed.

Third, all data were self-reported and may be subject to recall and social desirability bias, particularly in the practice domain, but standardized questionnaire administration, strict data quality control, and acceptable internal consistency of the scales were applied to reduce measurement error. Fourth, although the questionnaire showed acceptable overall internal consistency, the Cronbach’s α coefficient for the knowledge domain in the formal survey was 0.6199, which was lower than the conventional threshold of 0.70 and lower than that observed in the pilot test. This may be related to the dichotomous scoring format of the knowledge items and the greater heterogeneity of the formal survey sample. Therefore, the reliability of the knowledge domain should be interpreted cautiously, and further validation in larger and more diverse populations is warranted.

## Conclusion

In conclusion, patients receiving intravesical instillation demonstrated suboptimal disease-related knowledge, largely neutral attitudes, and positive practice behaviors, with health practices being primarily influenced by knowledge and attitudes rather than a sequential knowledge–attitude pathway. Proposed interventions should be linked to the specific item-level gaps identified in this study, including clarification of misconceptions about nonstandard alternatives and regimen duration, management of fear of recurrence and side-effect concerns, and training in simple coping behaviors such as breathing exercises and family communication. These item-informed strategies may help enhance sustained self-management and treatment adherence, particularly among patients with lower educational attainment. However, their feasibility, acceptability, and effectiveness should be further evaluated in future implementation studies.

## Data Availability

The original contributions presented in the study are included in the article/**Supplementary Material**. Further inquiries can be directed to the corresponding author/s.

## Supplementary material

Supplementary Figure 1 Measurement model of the Knowledge-Attitude-Practice (KAP) questionnaire. Ovals represent latent variables (Knowledge, Attitude, and Practice), and rectangles represent observed variables (questionnaire items k1–k15, a1–a12, and p1–p10). Single-headed arrows from latent to observed variables indicate factor loadings. ϵ1–ϵ37 denote the residual variances (measurement errors) for each corresponding observed variable. Double-headed arrows between latent variables represent covariances or correlations.

Supplementary Table 1 Criteria and examples for excluding outliers in baseline physical characteristics and disease recurrence variables.

Supplementary Table 2 Validity test of the total scale: KMO and Bartlett’s test of sphericity.

Supplementary Table 3 Confirmatory factor analysis model fit indices.

Supplementary Table 4 Confirmatory factor analysis path coefficients and factor loadings.

Supplementary Table 5 Distribution of knowledge dimension responses.

Supplementary Table 6 Distribution of attitude dimension responses.

Supplementary Table 7 Distribution of practice dimension responses.

Supplementary Table 8 SEM fit indicators.
