# Supplementary material for: Knowledge, attitudes, and practices related to health management among patients receiving intravesical instillation
Source: Front Oncol. 2026 Aug 3;16:1879500. doi: 10.3389/fonc.2026.1879500 (PMC13477935; doi:10.3389/fonc.2026.1879500)
Supplement: Supplementary file 1 [file DataSheet1.pdf]

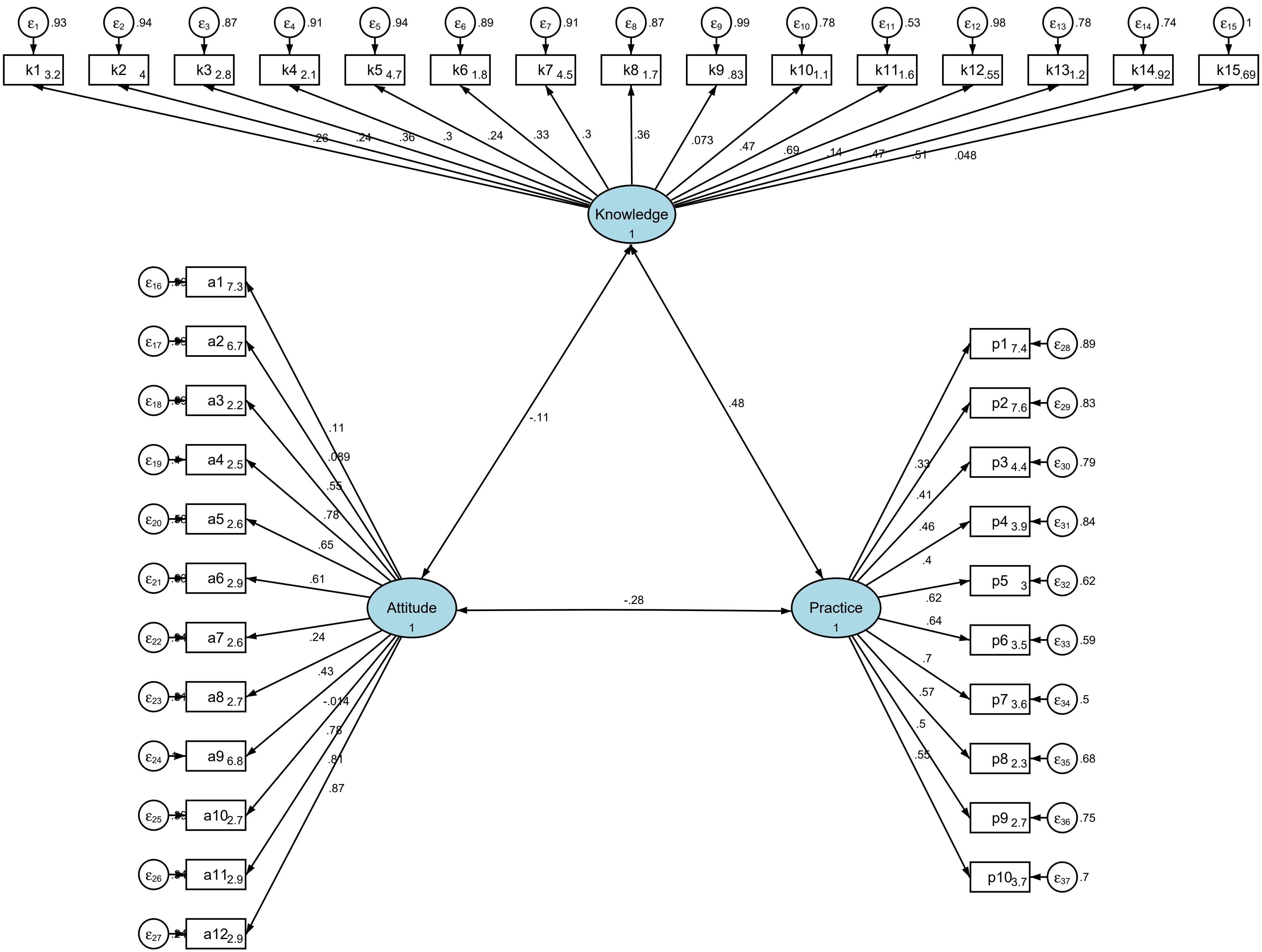

ε1 .93
ε2 .94
ε3 .87
ε4 .91
ε5 .94
ε6 .89
ε7 .91
ε8 .87
ε9 .99
ε10 .78
ε11 .53
ε12 .98
ε13 .78
ε14 .74
ε15 1
k1 3.2
k2 4
k3 2.8
k4 2.1
k5 4.7
k6 1.8
k7 4.5
k8 1.7
k9 .83
k10 1.1
k11 1.6
k12 .55
k13 1.2
k14 .92
k15 .69
.26
.24
.36
.3
.24
.33
.3
.36
.073
.47
.69
.14
.47
.51
.048
Knowledge
1
-.11
.48
ε16
a1 7.3
ε17
a2 6.7
ε18
a3 2.2
ε19
a4 2.5
ε20
a5 2.6
ε21
a6 2.9
ε22
a7 2.6
ε23
a8 2.7
ε24
a9 6.8
ε25
a10 2.7
ε26
a11 2.9
ε27
a12 2.9
.11
.089
.55
.78
.65
.61
.24
.43
-.014
.78
.81
.87
Attitude
1
-.28
Practice
1
p1 7.4
ε28 .89
p2 7.6
ε29 .83
p3 4.4
ε30 .79
p4 3.9
ε31 .84
p5 3
ε32 .62
p6 3.5
ε33 .59
p7 3.6
ε34 .5
p8 2.3
ε35 .68
p9 2.7
ε36 .75
p10 3.7
ε37 .7
.33
.41
.46
.4
.62
.64
.7
.57
.5
.55
