# Supplementary material for: Knowledge, attitudes, and practices related to health management among patients receiving intravesical instillation
Source: Front Oncol. 2026 Aug 3;16:1879500. doi: 10.3389/fonc.2026.1879500 (PMC13477935; doi:10.3389/fonc.2026.1879500)
Supplement: Supplementary file 2 [file Table2.docx]

**Supplementary table 1. Criteria and examples for excluding outliers in baseline physical characteristics and disease recurrence variables**

| **Variable Name** | **Criteria for Outlier Exclusion** | **Typical Examples of Outliers from the Screenshot** |
| --- | --- | --- |
| **Age** | Logical entry errors (Non-numeric / Obvious anomalies) | "Chen Duosheng", "No. 8", "Zai" *(Names/IDs accidentally entered instead of age)* |
| **Height (cm)** | Exclude values **< 100 cm** or **> 1000cm** | 1750, 16975, 17, 17065, 68 |
| **Weight (kg)** | Exclude values **< 30 kg** or **> 200 kg** | 5 *(Extremely underweight)*, 500 *(Extremely overweight)* |
| **Recurrence Count** | Logical entry errors / Non-numeric anomalies | Missing entries or incorrectly formatted blanks/characters in the final rows |

**Supplementary table 2. Validity test of the total scale: KMO and Bartlett’s test of sphericity**

| **Test Indicators** | Statistic Value | Degrees of Freedom (df) | p-value |
| --- | --- | --- | --- |
| KMO | 0.8118 |  |  |
| Bartlett's Test of Sphericity | 5408.293 | 666 | < 0.001 |

**Supplementary table 3. Confirmatory factor analysis model fit indices**

| **Indicators** | **Reference** | **Results** |
| --- | --- | --- |
| RMSEA | <0.080 | 0.073 |
| SRMR | <0.080 | 0.058 |
| TLI | >0.800 | 0.893 |
| CFI | >0.800 | 0.904 |

**Supplementary table 4. Confirmatory factor analysis path coefficients and factor loadings**

| Pathway | | Coefficient | std. err. | z | P>z | [95% conf. interval] | |
| --- | --- | --- | --- | --- | --- | --- | --- |
| k1 | Knowledge | 0.2585089 | 0.0545382 | 4.74 | <0.001 | 0.151616 | 0.3654017 |
| k2 | Knowledge | 0.23788 | 0.055347 | 4.3 | <0.001 | 0.1294018 | 0.3463583 |
| k3 | Knowledge | 0.3551135 | 0.0516519 | 6.88 | <0.001 | 0.2538776 | 0.4563494 |
| k4 | Knowledge | 0.2991482 | 0.0533179 | 5.61 | <0.001 | 0.1946471 | 0.4036494 |
| k5 | Knowledge | 0.2425783 | 0.0557092 | 4.35 | <0.001 | 0.1333903 | 0.3517663 |
| k6 | Knowledge | 0.3310442 | 0.0549905 | 6.02 | <0.001 | 0.2232648 | 0.4388236 |
| k7 | Knowledge | 0.2995025 | 0.0541361 | 5.53 | <0.001 | 0.1933977 | 0.4056073 |
| k8 | Knowledge | 0.3632319 | 0.056212 | 6.46 | <0.001 | 0.2530585 | 0.4734054 |
| k9 | Knowledge | 0.0730888 | 0.0613021 | 1.19 | 0.233 | -0.0470612 | 0.1932387 |
| k10 | Knowledge | 0.4671001 | 0.0494623 | 9.44 | <0.001 | 0.3701557 | 0.5640445 |
| k11 | Knowledge | 0.6884209 | 0.041957 | 16.41 | <0.001 | 0.6061867 | 0.7706551 |
| k12 | Knowledge | 0.1365707 | 0.0565287 | 2.42 | 0.016 | 0.0257764 | 0.247365 |
| k13 | Knowledge | 0.4653434 | 0.0516901 | 9 | <0.001 | 0.3640326 | 0.5666542 |
| k14 | Knowledge | 0.5114275 | 0.0489604 | 10.45 | <0.001 | 0.4154669 | 0.6073881 |
| k15 | Knowledge | 0.0475625 | 0.0629719 | 0.76 | 0.45 | -0.0758602 | 0.1709852 |
| a1 | Attitude | 0.1057865 | 0.0500025 | 2.12 | 0.034 | 0.0077834 | 0.2037895 |
| a2 | Attitude | 0.0889094 | 0.0509705 | 1.74 | 0.081 | -0.010991 | 0.1888098 |
| a3 | Attitude | 0.5526703 | 0.0365154 | 15.14 | <0.001 | 0.4811014 | 0.6242392 |
| a4 | Attitude | 0.7767237 | 0.0237883 | 32.65 | <0.001 | 0.7300995 | 0.8233478 |
| a5 | Attitude | 0.6496812 | 0.0323462 | 20.09 | <0.001 | 0.5862838 | 0.7130786 |
| a6 | Attitude | 0.6072432 | 0.0335432 | 18.1 | <0.001 | 0.5414997 | 0.6729867 |
| a7 | Attitude | 0.2381765 | 0.0491757 | 4.84 | <0.001 | 0.1417938 | 0.3345592 |
| a8 | Attitude | 0.4329556 | 0.0419887 | 10.31 | <0.001 | 0.3506594 | 0.5152519 |
| a9 | Attitude | -0.013715 | 0.0511947 | -0.27 | 0.789 | -0.1140548 | 0.0866248 |
| a10 | Attitude | 0.783666 | 0.0218041 | 35.94 | <0.001 | 0.7409307 | 0.8264014 |
| a11 | Attitude | 0.8118645 | 0.0205776 | 39.45 | <0.001 | 0.7715332 | 0.8521959 |
| a12 | Attitude | 0.8744164 | 0.0164129 | 53.28 | <0.001 | 0.8422478 | 0.906585 |
| p1 | Practice | 0.3294534 | 0.0510028 | 6.46 | <0.001 | 0.2294898 | 0.429417 |
| p2 | Practice | 0.4123261 | 0.0483166 | 8.53 | <0.001 | 0.3176272 | 0.5070249 |
| p3 | Practice | 0.4608077 | 0.0446612 | 10.32 | <0.001 | 0.3732734 | 0.5483419 |
| p4 | Practice | 0.3992786 | 0.047243 | 8.45 | <0.001 | 0.306684 | 0.4918733 |
| p5 | Practice | 0.6196364 | 0.0364449 | 17 | <0.001 | 0.5482056 | 0.6910672 |
| p6 | Practice | 0.6376888 | 0.0356936 | 17.87 | <0.001 | 0.5677307 | 0.7076469 |
| p7 | Practice | 0.7048322 | 0.0316216 | 22.29 | <0.001 | 0.642855 | 0.7668095 |
| p8 | Practice | 0.567795 | 0.0413409 | 13.73 | <0.001 | 0.4867684 | 0.6488216 |
| p9 | Practice | 0.5011551 | 0.0449285 | 11.15 | <0.001 | 0.4130969 | 0.5892133 |
| p10 | Practice | 0.5483554 | 0.0410409 | 13.36 | <0.001 | 0.4679167 | 0.6287941 |

**Supplementary table 5. Distribution of knowledge dimension responses**

| **Knowledge** | Correct | Incorrect | Uncertain |  |
| --- | --- | --- | --- | --- |
| 1. Intravesical chemotherapy can effectively prevent tumor recurrence. | 384 (91%) | 4 (0.95%) | 34 (8.06%) | |
| 2. After intravesical instillation, the drug solution should be retained in the bladder for at least 1 hour before urination. | 397 (94.08%) | 12 (2.84%) | 13 (3.08%) | |
| 3. Bladder irritation symptoms, such as urinary frequency, urgency, and dysuria, are common side effects of intravesical instillation. | 374 (88.63%) | 8 (1.9%) | 40 (9.48%) | |
| 4. Painless gross hematuria may occur in the early stage of recurrence. | 344 (81.52%) | 3 (0.71%) | 75 (17.77%) | |
| 5. Online health information should be verified with a physician to be considered reliable. | 404 (95.73%) | 3 (0.71%) | 15 (3.55%) | |
| 6. Depressive mood may affect treatment adherence. | 321 (76.07%) | 7 (1.66%) | 94 (22.27%) | |
| 7. Regular cystoscopic examinations are more important than self-monitoring of symptoms. | 402 (95.26%) | 3 (0.71%) | 17 (4.03%) | |
| 8. Smoking significantly increases the risk of recurrence. | 318 (75.36%) | 6 (1.42%) | 98 (23.22%) | |
| 9. Traditional Chinese medicine remedies can replace standard intravesical instillation therapy. | 27 (6.4%) | 172 (40.76%) | 223 (52.84%) | |
| 10. For high-risk patients, intravesical instillation should be continued for at least one year; for intermediate-risk patients, one year of intravesical instillation is recommended; for low-risk patients, a single immediate instillation may be sufficient. | 240 (56.87%) | 34 (8.06%) | 148 (35.07%) | |
| 11. Bacillus Calmette–Guérin (BCG) intravesical instillation is indicated for patients with intermediate- to high-risk non–muscle-invasive bladder cancer. | 302 (71.56%) | 15 (3.55%) | 105 (24.88%) | |
| 12. Gemcitabine intravesical instillation can be used in patients who fail BCG therapy. | 106 (25.12%) | 99 (23.46%) | 217 (51.42%) | |
| 13. Immunotherapy (BCG) is more likely to cause fever than chemotherapeutic agents. | 242 (57.35%) | 19 (4.5%) | 161 (38.15%) | |
| 14. The total duration of intravesical chemotherapy is usually shorter than that of BCG therapy (e.g., intermediate-risk patients receiving chemotherapy may require only 6–12 weeks, whereas BCG therapy requires maintenance for 1–3 years). | 194 (45.97%) | 13 (3.08%) | 215 (50.95%) | |
| 15. BCG instillation should be discontinued immediately if hematuria occurs during treatment. | 134 (31.75%) | 135 (31.99%) | 153 (36.26%) | |

**Supplementary table 6. Distribution of attitude dimension responses**

| **Attitude** | **Strongly agree** | **Agree** | **Neutral** | **Disagree** | **Strongly disagree** |
| --- | --- | --- | --- | --- | --- |
| 1. I believe that strictly following medical advice can reduce the risk of recurrence. | 311 (73.7%) | 93 (22.04%) | 12 (2.84%) | 3 (0.71%) | 3 (0.71%) |
| 2. Advice from doctors or nurses is more trustworthy than information obtained online. | 294 (69.67%) | 103 (24.41%) | 16 (3.79%) | 7 (1.66%) | 2 (0.47%) |
| 3. Concerns about side effects of intravesical instillation affect my willingness to continue long-term treatment. | 117 (27.73%) | 102 (24.17%) | 119 (28.2%) | 78 (18.48%) | 6 (1.42%) |
| 4. I often have difficulty sleeping because I worry about recurrence. | 67 (15.88%) | 100 (23.7%) | 121 (28.67%) | 119 (28.2%) | 15 (3.55%) |
| 5. Thinking about possible recurrence makes my heart race. | 61 (14.45%) | 98 (23.22%) | 123 (29.15%) | 123 (29.15%) | 17 (4.03%) |
| 6. Recurrence means that the treatment has completely failed. | 42 (9.95%) | 76 (18.01%) | 122 (28.91%) | 168 (39.81%) | 14 (3.32%) |
| 7. I often doubt the accuracy of online health information. | 60 (14.22%) | 100 (23.7%) | 145 (34.36%) | 105 (24.88%) | 12 (2.84%) |
| 8. Information on short-video platforms is easier to understand than that on professional medical websites. | 43 (10.19%) | 126 (29.86%) | 142 (33.65%) | 92 (21.8%) | 19 (4.5%) |
| 9. When encountering conflicting information, I tend to trust my doctor first. | 242 (57.35%) | 151 (35.78%) | 25 (5.92%) | 3 (0.71%) | 1 (0.24%) |
| 10. I have often felt low in mood over the past two weeks. | 48 (11.37%) | 106 (25.12%) | 138 (32.7%) | 111 (26.3%) | 19 (4.5%) |
| 11. This illness has made me lose confidence in the future. | 33 (7.82%) | 104 (24.64%) | 117 (27.73%) | 142 (33.65%) | 26 (6.16%) |
| 12. I feel unable to cope with the daily stress caused by the disease. | 42 (9.95%) | 92 (21.8%) | 141 (33.41%) | 132 (31.28%) | 15 (3.55%) |

**Supplementary table 7. Distribution of practice dimension responses**

| **Practice** | Always | Often | Sometimes | Rarely | Never |
| --- | --- | --- | --- | --- | --- |
| 1. Returning to the hospital on time for intravesical instillation. | 336 (79.62%) | 61 (14.45%) | 20 (4.74%) | 2 (0.47%) | 3 (0.71%) |
| 2. Strictly controlling fluid intake according to medical advice. | 306 (72.51%) | 97 (22.99%) | 14 (3.32%) | 4 (0.95%) | 1 (0.24%) |
| 3. Actively recording adverse reactions and reporting them in a timely manner. | 225 (53.32%) | 124 (29.38%) | 42 (9.95%) | 25 (5.92%) | 6 (1.42%) |
| 4. Undergoing regular cystoscopic follow-up examinations. | 229 (54.27%) | 93 (22.04%) | 66 (15.64%) | 20 (4.74%) | 14 (3.32%) |
| 5. Verifying online health information through multiple sources. | 145 (34.36%) | 125 (29.62%) | 74 (17.54%) | 49 (11.61%) | 29 (6.87%) |
| 6. Actively discussing information I have seen with my doctor. | 139 (32.94%) | 131 (31.04%) | 99 (23.46%) | 39 (9.24%) | 14 (3.32%) |
| 7. Obtaining disease-related knowledge through official or accredited platforms. | 146 (34.6%) | 147 (34.83%) | 81 (19.19%) | 34 (8.06%) | 14 (3.32%) |
| 8. Relieving anxiety through deep-breathing exercises. | 86 (20.38%) | 98 (23.22%) | 95 (22.51%) | 79 (18.72%) | 64 (15.17%) |
| 9. Sharing treatment-related stress with family members. | 75 (17.77%) | 106 (25.12%) | 111 (26.3%) | 100 (23.7%) | 30 (7.11%) |
| 10. Reducing psychological stress by adjusting my perspective on the disease and focusing on positive aspects. | 103 (24.41%) | 166 (39.34%) | 99 (23.46%) | 45 (10.66%) | 9 (2.13%) |

**Supplementary table 8. Structural equation model fit indicators**

| **Indicators** | **Reference** | **Results** |
| --- | --- | --- |
| RMSEA | <0.080 | 0.000 |
| SRMR | <0.080 | 0.000 |
| TLI | >0.800 | 1.000 |
| CFI | >0.800 | 1.000 |
